# Supplementary material for: Mobile phone use is associated with higher smallholder agricultural productivity in Tanzania, East Africa
Source: PLoS One. 2020 Aug 6;15(8):e0237337. doi: 10.1371/journal.pone.0237337 (PMC7410319; doi:10.1371/journal.pone.0237337)
Supplement: Attachment — Submitted filename: Review of PONE-D-20-05740.pdf [file pone.0237337.s002.pdf]

Review of PONE-D-20-05740

Mobile phone use associated with higher smallholder agricultural productivity in Tanzania, East Africa

This article seeks to "examine relationships between mobile phone use and agricultural productivity at the household and farmer levels" in Tanzania. Authors utilize multi-level modeling and principal component analysis to examine the nature and type of relationships between farmers' perception and mobile phone use in agriculture, and the level of agricultural productivity, measured by maize yields per hectare. There are three findings: First, a positive relationship between mobile utilization and agricultural yields. Second, a positive relationship between farmer-perception of mobile phones and "agricultural efficiency" in terms of lower costs, higher profits, and lower "time investments in farming". The third finding is that there is "no-consistent association between general phone use and self-reported maize yield" (in terms of the number of contacts, SMS sent and received, and calls made and received in short intervals).

Based on these findings, authors proposed policy recommendations including support for intentional use of mobile phones for agricultural production and the relevance of farmers' perception about mobile phone usage in agriculture. Their main conclusion is that there are important policy prospects that can help leverage current ICT usage to upsurge the extent of agricultural gains associated with mobile use in developing countries.

General observation

The topic is nice and can be relevant in advancing an understanding of the constraints to adoption of agricultural technologies like ICTs and other critical technologies that contribute to the sustainable development goals (SDGs) especially hunger and climate change. However, the current version of the paper needs substantial improvement or a complete overhaul if it is to be acceptable for publication. Furthermore, authors should let a non-author read the paper for consistency checks before making any resubmission.

Major comments

A. Writing, narrative, and structure

1. The paper is not well written. It lacks a coherent structure, which makes it hard for me to see what the authors are really trying to achieve.
2. I do not see a compelling contribution to literature. The potential contribution mentioned on pg. 5 (lines 111-120) has some problems including the endogeneity of perception due to its subjectivity, which the authors do not seem to address.
    - Authors do not clearly articulate the study's relevance beyond the setting.
    - The proposed policy contributions on pg. 6 (lines 135-143) appear more like study objectives than actual policy contributions. Better writing can be helpful here.
3. Insufficient motivation for use perception as the key policy variable in this study. Why should we care about the perception of farmers in the use of mobile phones for agriculture when the technology is not new?

- What is novel about mobile phone utilization in agriculture in the region, elsewhere in Africa, and beyond? I would like to see a more convincing justification on this subject and the use of perception as a policy var. Perception, in fact, is a major determinant of technology adoption and/or program participation in many contexts.
- I don't see what is new about farmers' perception of mobile phone use for agriculture in this context.

4. Another critical problem is how agricultural productivity is measured – crop yields are not the only measure of productivity.
   - Self-reported yield estimates are weak due to several associated errors.
   - What actions did you take to account for this risk? See relevant example papers on this critical topic (Amadu et al., 2020; Desiere and Jolliffe, 2018; Godlonton et al., 2017; Judge and Schechter, 2009; Wossen et al., 2019).
5. Insufficient description of the study area and data. Figure 1, the google earth map, is blurry. Please use an actual GIS map with coordinates to show your study sites (e.g., Coulibaly et al., 2017; Rana, and Miller, 2019; Van Campenhout, 2017). In general, all the Figures are blurry and unacceptable.

B. Data, conceptual framework and variables

1. There is no conceptual framework for the paper. Authors can do a better job by presenting a conceptual framework that guides the statistical operations in the paper. This is critical for any quantitative analysis.
2. Therefore, the "Data analysis" section (pg. 10+) does not make much sense to me. For instance, how are the key variables measured like perception, various uses of mobile in relation to agriculture? See for example (Amadu et al., 2020; Rana, and Miller, 2019).
3. Therefore, the two models presented on pg. 11 (lines 254 & 255) and not clear.
4. Paper lacks a convincing presentation of data. I kept revising the paper to see:
   - how can I understand and interpret the data and results (albeit not really a result, but descriptive stats – more on this below).
   - For example, on pg. 9 (lines 193 – 196), I am confused as to whether some household members did not undertake farming? Is this articulated in the narrative? If yes, where? If no, why not?
5. Moreover, I suggest you provide a "theoretical expectations" section to guide the interpretation of the key variables based on sound literature review. This is lacking and need attention.
   - For example, you may want to caption part of pg. 12 to 13 as either literature review, or theoretical expectation, instead of making statements like "these covariates were informed by literature…" (pg. 13, L. 286).
   - We expect literature to inform your work. Therefore, just present a section to discuss the main vars and covariates in terms of what obtains elsewhere and expectations in this setting.
6. Following the above points, there should been a summary stats' table upfront to show the mean of all variables in the analysis.

7. On pg. 9, writing on lines 198 to 206 is wordy. Consider using concise language for clarity. For e.g., use pretesting of questionnaires, instead of "conducted a practice survey …" (lines 202-203). On the same pg., (lines 209-212), delete sentence starting with "All respondents … study area". It is wordy and adds little.

C. Results, discussion, policy, limitation, and conclusion

1. The presentation of your results seems clumsy. For instance, your "results" (pg. 14) look more like descriptive statistics. Thus, I think Table 1 should be labelled 'summary statistics' rather than "results based on …". These are not rigorous analytical results.
2. Some variables in Table 1 had not been discussed in the narrative of the paper – the more reason you need to have a theoretical expectations section above. For example, "good year" is mentioned on pg. 11 (line 241) but not defined. "Bad year" is not mentioned earlier at all. Likewise, "synthetic phone" had not been defined earlier in the narrative, except for the mention of "synthetic variables" on pg. 12 (line 271).
3. Table 1 and all Tables should have notes immediately below, not above in the Table title.
4. Table 2 does not have a good title. Consider presenting the title of a Table as a statement like 'proportion of respondents using phones." Moreover, there should be a note under the Table to provide clarity. The table is not clear. Is there a column for the interaction of these variables such as using phones to discuss with friends and for selling crops? The Table does not present a complete picture.
5. Table 3 is not clear. Same points as above such as proper labelling and notes.
6. The "discussion" section (pg. 20 – 22) reads more like a results section.
7. On pg. 24, "intensity and frequency" appear for the first time in the paper. Why?
   - These are loaded terms, which should have been described in introduction or study area description before using them anywhere else.
   - Or they should have been conceptualized/operationalized in this study and then included in the analysis before using them here in the "limitations…" section.
8. I like your discussion of potential mechanisms (pg. 21) through which mobile phones may lead to yield enhancement. However, you do not provide statistical analyses for any of these mechanisms in the paper to bolster your findings (see. For example, Van Campenhout, 2017).
9. Following the above point, there should be a robustness check for your findings.
10. The conclusion is too terse and makes no sense. Should be succinct but convey enough information to appear as 'stand-alone' for an impatient reader. For example, the starting sentence (pg. 26 line 518) is awkward. In short, I would not consider this section as a conclusion for the paper because it presents little or nothing about the rest of the paper.

Other/Minor comments

1. Be consistent about the use of sub-Saharan Africa versus Africa. To maintain the flow, choose one and stick to it, or you can indicate your intentional inter-use at the beginning of the paper and move on.
2. Introduction is too long being 4 pages. Reduce to 2 or 2.5 pages.
3. On pg. 5 (line 108) remove many before fewer studies.

4. On pg. 6 (line 140-143), delete sentence starting with “Thus… and ending with productivity”. That whole sentence is not only poorly written but does not really add much to the argument of the paper.
5. On pg. 7 (L. 156) insert ‘distance from’ after “in relation to” …
6. Also, on pg. 7, delete from lines 167 – 168. It adds nothing.
7. On pg. 8 (L. 171), write Data (delete collection). On line 173, write ‘Data for this study come from before “Focus group…”
8. Pg. 9: On L. 193, delete “both a”. On L. 194, write members.
9. On pg. 12, line 266 is missing something. Following line 255, I expected the two models.
10. The entire paper can be significantly enhanced if the authors can clean up typos.

References

Amadu, F. O., Miller, D. C., McNamara, P. E., 2020. Yield effects of climate-smart agriculture aid investments in southern Malawi. *Food Policy. 92*, 101869. https://authors.elsevier.com/a/1ay3A15oGp6Skf.

Coulibaly, J.Y., Chiputwa, B., Nakelse, T. and Kundhlande, G., 2017. Adoption of agroforestry and its impact on household food security among farmers in Malawi. *Agricultural Systems,* 155**,** 52-69.

Desiere, S., Jolliffe, D., 2018. Land productivity and plot size: Is measurement error driving the inverse relationship? *Journal of Development Economics. 130,* 84–98. https://doi.org/10.1016/j.jdeveco.2017.10.002.

Godlonton, S., Hernandez, M. A., Murphy, M., 2017. Anchoring Bias in recall data: Evidence from Central America. *Amer. J. Agr. Econ. 0(0),* 1–23; doi: 10.1093/ajae/aax080.

Judge, G., Schechter, L., 2009. Detecting Problems in Survey Data Using Benford's Law. *Journal of Human Resources* 44 (1) 1-24.

Rana, P., Miller, D. C., 2019. Explaining longterm outcome trajectories in social–ecological systems. PLoS ONE 14(4): e0215230. https://doi.org/10.1371/journal.pone.0215230.

Van Campenhout, Bjorn., 2017. There is an app for that? The impact of community knowledge workers in Uganda, Information, Communication & Society, 20:4, 530-550, DOI: 10.1080/1369118X.2016.1200644

Wossen, T., Alene, A., Abdoulaye, T., Feleke, S., Manyong, V., 2019. Agricultural technology adoption and household welfare: Measurement and evidence. *Food Policy 87, 101742.* https://doi.org/10.1016/j.foodpol.2019.101742.
