# Supplementary material for: Mobile phone use is associated with higher smallholder agricultural productivity in Tanzania, East Africa
Source: PLoS One. 2020 Aug 6;15(8):e0237337. doi: 10.1371/journal.pone.0237337 (PMC7410319; doi:10.1371/journal.pone.0237337)
Supplement: Attachment — Submitted filename: Review comments _05740.docx [file pone.0237337.s001.docx]

Review comments

**Manuscript ID** – PONE – D – 20 – 05740: **Title:** Mobile Phone use Associated with Higher Smallholder Agricultural Productivity in Tanzania – East Africa. **Due date:** 3^rd^ April 2020

General Comments:

Good focus of the paper and with a clear new novelty (Perceptions on MP use versus agricultural productivity. Well written and referenced but a few technical comments highlighted below if addressed would make the paper better.

Specific Comments:

TITLE:

1. I think there should be a “,” and “is” between “use” and “associated”

ABSTRACT

2. Generally comprehensive and easy to understand
3. there should be some highlights on the data sample size used,
4. and methods used to analyze the data should also be concisely highlighted in the abstract

INTRODUCTION

1. Generally well written, referenced and easy to understand.
2. However – villages of interest in Tanzania are not mentioned at all, or
3. Perhaps some specific statistics of this Iringa province, since all statistics mentioned here are only at regional or government level. Provincial or village specific stats, would make the write up stronger.
4. Novelty is clear that, the paper focuses on farmers’ perceptions on agricultural productivity, which has rarely been researched
5. A good mix of qualitative and quantitative approach

METHODS

1. Some specific statistics about the four villages – would be interesting to look at here briefly
2. Line 178 – what is meant by “household diversity?” Technically this is largely used with regards to household production diversity or household dietary diversity, so it may be confusing here – and you may need another term to avert the potential confusion to readers
3. Line 187 – what informed the sample size of 279? Some background could be helpful
4. Line 204 – how was it likely to influence the responses by having same gender enumerator/respondents? This needs to be explained as it is not usual, and would be a potential source of biases
5. Lines 288 – 290 – variables age and education based on several literatures can influence mobile phone use, as well as agricultural yield. Moreover, they also make logical sense to be controlled for other than being merely excluded because they were not significant. This would deny us potentially logical and economic sense/significance based modelling which is another excellent component for validity of results on top of statistical significance. I would wish to see how the model results come out with these logical, and economically valid variables included
6. Also, a wealth index and use of mobile phones, could be endogenous; how was this potential endogeneity handled?
7. Line 295 – use either 1 to 100 or one to a hundred – to avert potential confusion
8. Line 296 – was farm-size transformed because it was not normal? If yes, this has to be stated, but not only stated for a few variables
9. Lines 309 – 311 – the assumption of number of calls or SMSs reflecting long time use of the phone; did you control for the seasonality aspect? I would assume that during the planting or harvesting seasons, phone calls would be more. Perhaps, you may need to clarify on the seasonality aspect with regards to this assumption

RESULTS

1. Table 1; were these statistics different across the 4 villages? It would be nice to show these. Also, in discussions of these results, it would be good to briefly show how these compare with national statistics or regional or provincial ones.
2. In Table 2, presenting results in percentages would make it easier for comparison and understanding
3. Table 3 – for ease of understanding results in line with usual literature – could you show the significance levels of each of the control variables by asterisk? Also reduce the grid lines in the table for more neatness.
4. Age and Education should also be controlled for in the results of this table 3 for their potentially significant logical and economic importance.

DISCUSSIONS

1. Line 404 – could you be specific with these confounding factors that contrary to general literature – led the results of general MP use not to be associated significantly with yield? This would make the paper more “self-contained” and independent
2. In Policy Implications – perhaps you must strongly make it clear that this advise is intended to “smallholder farmers” who are investigated in this paper. Otherwise – blindly advising policy to generally support MP use on specific agricultural activities may not have similar results for cattle herdsmen who are also common in rural Tanzania.

CONCLUSIONS

1. There was only about 4% of the sample using smart phones – and why make this a priority concluding remark in line 528 – in the first real paragraph that should be aimed at the central results of the study.
